# Revisiting the Coreceptor Function of Complement Receptor Type 2 (CR2, CD21); Coengagement With the B-Cell Receptor Inhibits the Activation, Proliferation, and Antibody Production of Human B Cells

**DOI:** 10.3389/fimmu.2021.620427

**Published:** 2021-04-01

**Authors:** Kristóf G. Kovács, Bernadett Mácsik-Valent, János Matkó, Zsuzsa Bajtay, Anna Erdei

**Affiliations:** ^1^Department of Immunology, Eötvös Loránd University, Budapest, Hungary; ^2^MTA-ELTE Immunology Research Group, Eötvös Loránd University, Budapest, Hungary

**Keywords:** B cell, CR2 (CD21), coreceptor, Ca^2+^ response, inhibition, proliferation, activation marker, cytokine and Ig-production

## Abstract

The positive coreceptor function of complement receptor type 2 [CR2 (CD21)] on B cells is generally accepted, although its role in the enhancement of antibody production had only been proven in mice. The importance of this phenomenon prompted reinvestigation of the functional consequences of coclustering CD21 and the B cell receptor (BCR) on primary human cells. We found that, at non-stimulatory concentrations of anti-IgG/A/M, coclustering the BCR and CR2 enhanced the Ca^2+^ response, while activation marker expression, cytokine production, proliferation, and antibody production were all inhibited upon the coengagement of CR2 and BCR on human B cells. Thus, the “textbook dogma” claiming that C3d acts as an adjuvant to enhance humoral immunity is relevant only to mice and not to humans.

## Introduction

It is generally accepted that complement receptor type 2 [CR2 (CD21)] is an activating coreceptor for B cells despite the fact that the enhancement of antibody production upon its coclustering with the B cell receptor (BCR) has only been proven in murine systems [extensively reviewed in (1)] but not in humans. Furthermore, while mouse CR1 (CD35) possesses the same C-terminal sequence and exerts a similar coreceptor role as CR2, human CR1 has been demonstrated to possess a different structure and inhibit several functions of B lymphocytes, including proliferation and antibody production (2–8). Therefore, the “textbook dogma” claiming that antigen-bound C3-fragments, that is ligands for CR1 and CR2, act as a molecular adjuvant cannot be generalized from one species to another.

The marked difference between the function of CR1 and CR2 in human and in mice is defined by the differences in the genetic background and the structure of these receptors (9). In mice, CR1 and CR2 (often designated CR1/2) are encoded by a single gene (*Cr2*), and alternative splicing results in CR2, the shorter form of the receptor. In contrast to this, human CR1 and CR2 are encoded by two distinct genes (*CR1* and *CR2*), which encode receptors with diverse ligand-specificities and different functions.

CR2 is a 145 kDa transmembrane glycoprotein, which is mainly expressed by B lymphocytes and follicular dendritic cells (10, 11). It binds complement C3-derived fragments C3d, C3dg and with lower affinity iC3b deposited on antigens (12, 13). The extracellular part of human CR2 consists of 15 or 16 short consensus repeat (SCR) domains (9). In its intracellular region it comprises a 34-residue tail, which denotes that CR2 may require other membrane proteins to induce signal transduction both in men and mice. CD21 has been proven to form a signaling complex with CD19, CD81, and Leu-13 in B cells (14, 15), however Tuveson et al. demonstrated that in the membrane of human B lymphocytes CD21 also appears in complex with CD35, when the association between CD21 and CD19 is prevented (16).

Human CR1 is ~200 kDa glycoprotein that binds C3b/C4b and iC3b complement fragments. In addition to its inhibitory role in human BCR-driven functions (2–8), human CR1 serves as a cofactor in factor I-mediated cleavage of C3b/C4b and accelerates the decay of C3 and C5 convertases (17). This multifunctional protein is expressed by several cell types, including erythrocytes, phagocytes, and B cells (18). The most common isoform of CR1 is composed of 30 SCRs. Its cytoplasmic tail of 43 residues contains a six-amino-acid sequence that is homologous to the sequence in the epidermal growth factor receptor, phosphorylated by protein kinase C (19). The inhibitory role of ligated CR1 in BCR-driven intracellular events in human B cells was proven by Kremlitzka et al. by demonstrating lower phosphorylation of signaling molecules (6).

We have been investigating the effect of C3-derived ligands on B cell functions both in human and mice for several years [reviewed recently in (20)]. Studying human B cells, however, we have never observed an enhanced antibody production upon coengagement of CR2 and BCR. This observation is strengthened by the fact that no data proving the coreceptor function of CD21 in antibody production by human B cells are published. Despite the lack of evidence, it is generally accepted that CR2 is a positive regulator of BCR induced antibody production in human, as well. Regrettably, most reviews ignore published data highlighting major differences regarding the genetic background and the function of CR1 and CR2 between humans and mice. To clarify whether the “textbook dogma” describing CR2 as a positive coreceptor of BCR does apply to human systems, we decided to reinvestigate this important, but controversial, issue.

We employed well-defined experimental conditions and studied the effect of coclustering BCR and CR2 on the release of intracellular free Ca^2+^ and the expression of an early activation marker, cytokine release, proliferation, and antibody production by human B lymphocytes.

## Materials and Methods

### Cells

Tonsils of children undergoing routine tonsillectomies at Saint István Hospital were used in compliance with the Declaration of Helsinki. The study was approved by the Ethics Committee of the Medical Research Council in Hungary (TUKEB; 52088/2015/EKU). Mononuclear cells were isolated from the tonsils by applying Ficoll-Paque gradient centrifugation (GE Healthcare, Chicago, IL, USA). T cells were removed by rosetting mononuclear cells with 2-aminoethylisothiouronium bromide (AET; Sigma-Aldrich, St. Louis, MO, USA) -treated sheep red blood cells using Ficoll-Paque centrifugation. Resting B cells were obtained by centrifugation on Percoll gradient (Sigma-Aldrich, St. Louis, MO, USA). The isolation efficiency yielded above 95% in each case, which was verified by using a FACSCalibur flow cytometer (Becton-Dickinson Biosciences, San Jose, CA, USA), through FITC-conjugated mouse anti-human CD19 monoclonal antibody staining (ImmunoTools, Friesoythe, Germany) for 30 min at 4°C and using CellQuest Pro software (Becton-Dickinson Biosciences).

### Preparation of Preformed Complexes

Proteins were biotinylated using N-hydroxysuccinimidobiotin (NHS-biotin; H1759; Sigma-Aldrich), following the instructions of the manufacturer. Complexes consisting of various concentrations of biotinylated AffiniPure *F*(ab′)_2_ Fragment Goat Anti-Human immunoglobulin G (IgG)/A/M (Jackson ImmunoResearch, Cambridgeshire, UK) and biotinylated human C3d (Sigma-Aldrich) were mixed with 2.1 μg/ml streptavidin (Thermo Fisher Scientific, Waltham, MA, USA) before the experiments, as described by Buhlmann et al. (21). The suboptimal concentration of anti-IgG/A/M was determined in preliminary experiments.

### Culture Conditions

High-density, resting tonsillar B cells were cultured in the presence of different stimuli in RPMI-1640 medium (Sigma-Aldrich) containing 10% FCS (Thermo Fisher Scientific) and 50 μg/ml gentamycin (Sigma-Aldrich) at 37°C and 5% CO_2_. For assessing the proliferative capacity of the cells, 96-well flat bottom culture plates (Corning, New York, USA), while round bottom culture plates (Corning) were used for monitoring activation marker expression, cytokine release, and antibody production.

### Release of Intracellular Free Ca^2+^

Calcium indicator Fluo-4 AM (Thermo Fisher Scientific, Waltham, MA, USA) was loaded into 1 × 10^7^ resting B cells, following the instructions of the manufacturers. 7-amino-actinomycin D (7-AAD; Becton-Dickinson Biosciences) staining was applied to exclude non-viable cells. Samples of 5 × 10^5^ cells had been kept on ice in a culture medium until the measurements were carried out at 37°C. The fluorescence intensity of Fluo-4, indicating the concentration of intracellular free Ca^2+^ ([Ca^2+^]_i_), was monitored for 300 s by flow cytometry on FACSCalibur instrument using CellQuest Pro software. After baseline registration for 15 s, the samples were treated with various concentrations of the preformed complexes, as indicated. The Ca^2+^ response kinetics and the percentage of B cells responding with elevated fluorescence intensity upon treatments were examined with FlowJo 10 software (FlowJo, Ashland, OR, USA) and illustrated with GraphPad Prism 6 software (GraphPad Software, San Diego, CA, USA). Statistical analysis of the peak amplitudes of the Ca^2+^ response curves was utilized by GraphPad Prism with the one-way analysis of variance (ANOVA). The mean peak amplitudes ± SD of distinctly treated samples were compared to the peak amplitude of control samples taken as 100%, which was obtained in three independent experiments. Images were prepared with PaintShop Pro 6 software (Jasc Software, Eden Prairie, MN, USA).

### Activation Marker Expression

4 × 10^5^ resting B cells/well were cultured in 96-well microplates in the presence of the preformed complexes. The cells were stained with FITC-conjugated mouse anti-human CD69 (Becton-Dickinson Biosciences, San Jose, CA, USA) after 24 h for 30 min at 4°C. The expression of the activation marker was measured using a CytoFLEX flow cytometer (Beckman Coulter, Brea, CA, USA) with CytExpert 2 software (Beckman Coulter). Non-viable cells were excluded by propidium iodide staining (Thermo Fisher Scientific). Histograms were prepared using the Kaluza Analysis Software 2.1 (Beckman Coulter). The geometric mean fluorescence intensity (gMFI) values were further analyzed statistically by GraphPad Prism using the one-way ANOVA. To analyze the changes of CD69 expression, the gMFI values of untreated samples were subtracted from the gMFI values of treated samples, and the mean ± SD of treated samples was compared to the control samples taken as 100%. Images were prepared with PaintShop Pro.

### Cytokine Production

4 × 10^5^ resting B cells/well were cultured in 96-well microplates with a 100-μl culture medium containing the preformed complexes in concentrations shown. After 48 h incubation, the interleukin (IL)-6 content of the supernatants was determined using ELISA (Sino Biological, Beijing, China) by following the instructions of manufacturers. The mean IL-6 concentrations ± SD of treated samples were compared to the IL-6 concentration of control samples taken as 100%. Images were prepared with PaintShop Pro.

### Proliferation

4 × 10^5^ B cells/well were placed in a 100-μl culture medium containing different concentrations of the preformed complexes, as shown in the figures. After 48 h, the cells were pulsed with 1 μCi/well [^3^H]-thymidine (NEN, Boston, MA, USA) for 18 h. Incorporated radioactivity was measured with a Wallac 1409 liquid scintillation beta counter (Wallac, Allerød, Denmark). The mean counts per minute (cpm) values ± SD of treated samples were compared to the cpm values of control samples taken as 100%. Images were prepared with PaintShop Pro.

### Antibody Production

IgM and IgG enzyme-linked immunosorbent spot (ELISpot) assays (MabTech, Nacka Strand, Sweden) were performed following the instructions of the manufacturers. Briefly, 96-well polyvinylidene difluoride (PVDF) membrane plates (Millipore, Burlington, MA, USA) were coated with mouse anti-human IgM and IgG monoclonal antibodies (MabTech). Meanwhile, 3.2 × 10^5^ resting B cells/well were seeded in 96-well round bottom microplates and incubated in the presence of the preformed complexes and 50 ng/ml IL-2, IL-6, and IL-10 (ImmunoTools, Friesoythe, Germany) at 37°C and 5% CO_2_ for 72 h. Then, 5 × 10^4^ differently treated cells were placed into the wells of the PVDF plates and incubated at 37°C and 5% CO_2_ for 20 h. Then, biotinylated mouse anti-human IgM and IgG (MabTech, Nacka Strand, Sweden), streptavidin-conjugated HRP (MabTech), and TMB (MabTech) were added to the samples. B cells secreting IgM and IgG were assessed by an ImmunoSpot Reader (Cellular Technology, Shaker Heights, OH, USA) using ImmunoSpot 3 software (Cellular Technology). Images were prepared with PaintShop Pro.

### Statistical Analysis

All statistical analyses were performed using one-way ANOVA with the Dunnett's multiple comparison *post*-*hoc* test by GraphPad Prism. *P* < 0.05 values were considered significant.

## Results

### Co-ligation of BCR With CR2 Increases the Ca^2+^ Response of Human B Cells at Suboptimal BCR Stimulus

A previous study demonstrated that the lack of Ca^2+^ response in human B cells to a substimulatory dose of anti-IgM can be restored by co-crosslinking BCR and CR2 (21). In these experiments, Buhlmann et al. employed biotin-tagged anti-IgM and C3d, which were clustered *via* streptavidin on the cell surface.

To further examine and broaden these findings, we studied the Ca^2+^ response of human B cells activated by biotin-labeled ligands, namely the *F*(ab′)_2_ fragment of anti-IgG/A/M and C3d, the natural ligand of CR2 in various concentrations. The biotinylated ligands were crosslinked with 2.1 μg/ml streptavidin, and the preformed complexes were added to the resting human B cells.

**Figure 1:**
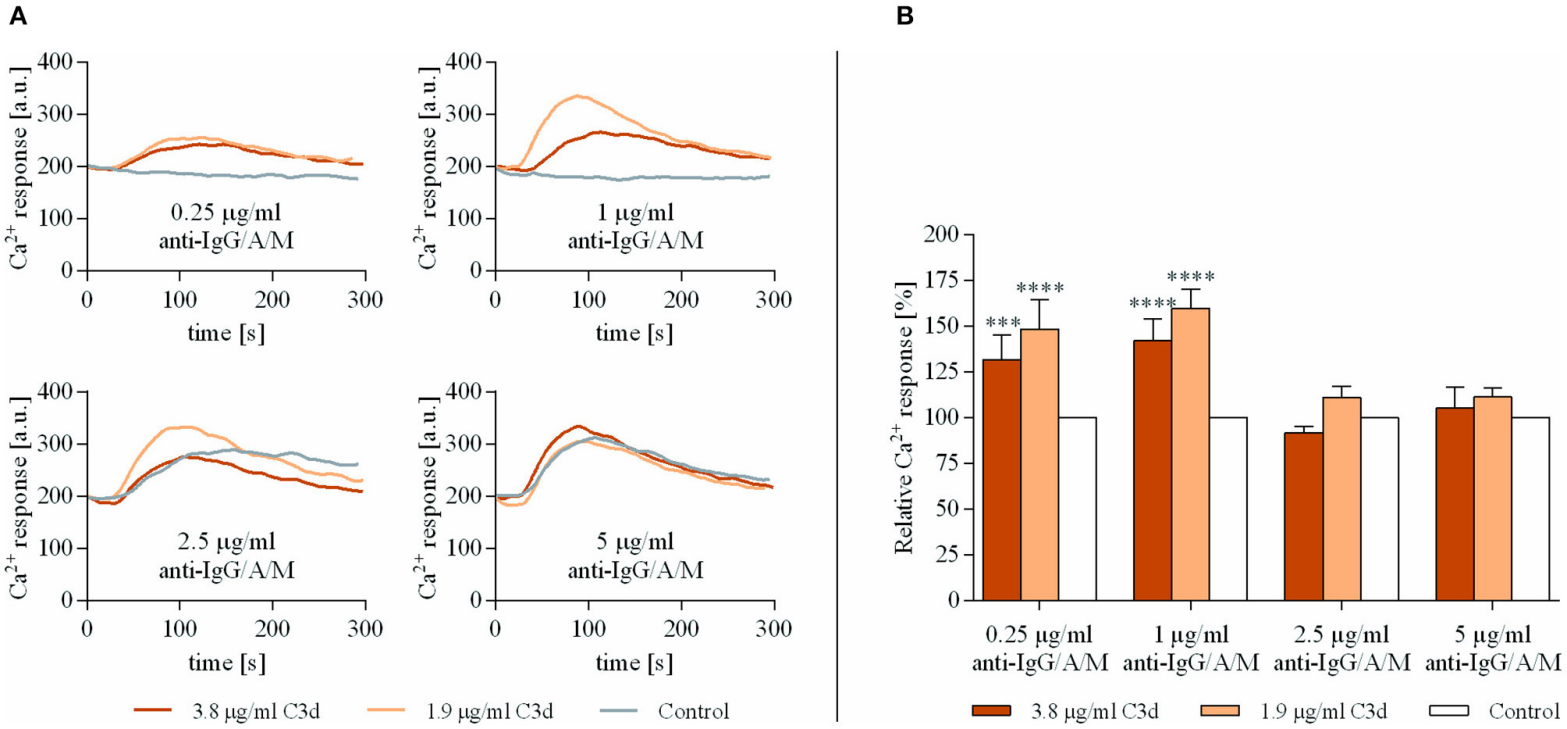
Co-ligation of complement receptor type 2 (CR2) with suboptimally stimulated B-cell receptor (BCR) elevates the Ca^2+^ response of human B cells. Resting human B cells were treated with complexes consisting of either streptavidin-conjugated anti-immunoglobulin G (IgG)/A/M (control samples) or streptavidin-linked anti-IgG/A/M and C3d molecules in different concentrations as indicated. Changes in fluorescence intensity were assessed for 300 s *via* flow cytometry and the Ca^2+^ response kinetics were examined. Response curves demonstrate the result of one representative experiment depicting mean fluorescence intensity as a function of time **(A)**. The peak amplitude of each Ca^2+^ response curve was presented as a relative value compared to the peak amplitude of the control samples. Diagrams show cumulative results obtained in three independent experiments displaying the mean of the relative values ± SD **(B)**. ***p < 0.001, ****p < 0.0001.

As shown in Figure 1, very low concentrations of anti-IgG/A/M (0.25 and 1 μg/ml), when added alone, did not exert any response, while coclustering BCR and CR2 caused a significantly enhanced Ca^2+^ response. This observation is in accordance with the findings of others (21, 22) and proved that the preformed complexes used in this study were biologically effective. On the other hand, at an optimal BCR stimulus (5 μg/ml anti-IgG/A/M), CR2-mediated enhancement of the intracellular free Ca^2+^ completely vanished. The analysis of the percentage of responding B cells revealed that at a suboptimal concentration of anti-IgG/A/M binding of C3d was not only capable of inducing a stronger Ca^2+^ response, but it also affected a higher percentage of B cells (Supplementary Figure 1). Interestingly, under conditions of suboptimal BCR stimulus, a lower concentration of the complement-derived ligand caused a stronger rise in intracellular free Ca^2+^ than the higher one, most probably due to the distribution and stoichiometry of CR2 and BCR on the surface of B cells.

These data show that simultaneous ligand binding of BCR and CR2 enhances the rise in intracellular free Ca^2+^ concentration, only under conditions when the antigen-binding receptor receives a suboptimal stimulus.

### C3d Inhibits the BCR Induced Expression of CD69

Next, we aimed to unveil the effects of co-ligation of CR2 and BCR on the expression of CD69, one of the very first phenotypic indicators of activation. To this end, resting human B cells were cultured in the presence of the complexes containing anti-IgG/A/M and C3d molecules. The expression of CD69 activation marker molecules was detected by flow cytometry after 24 h of incubation.

**Figure 2:**
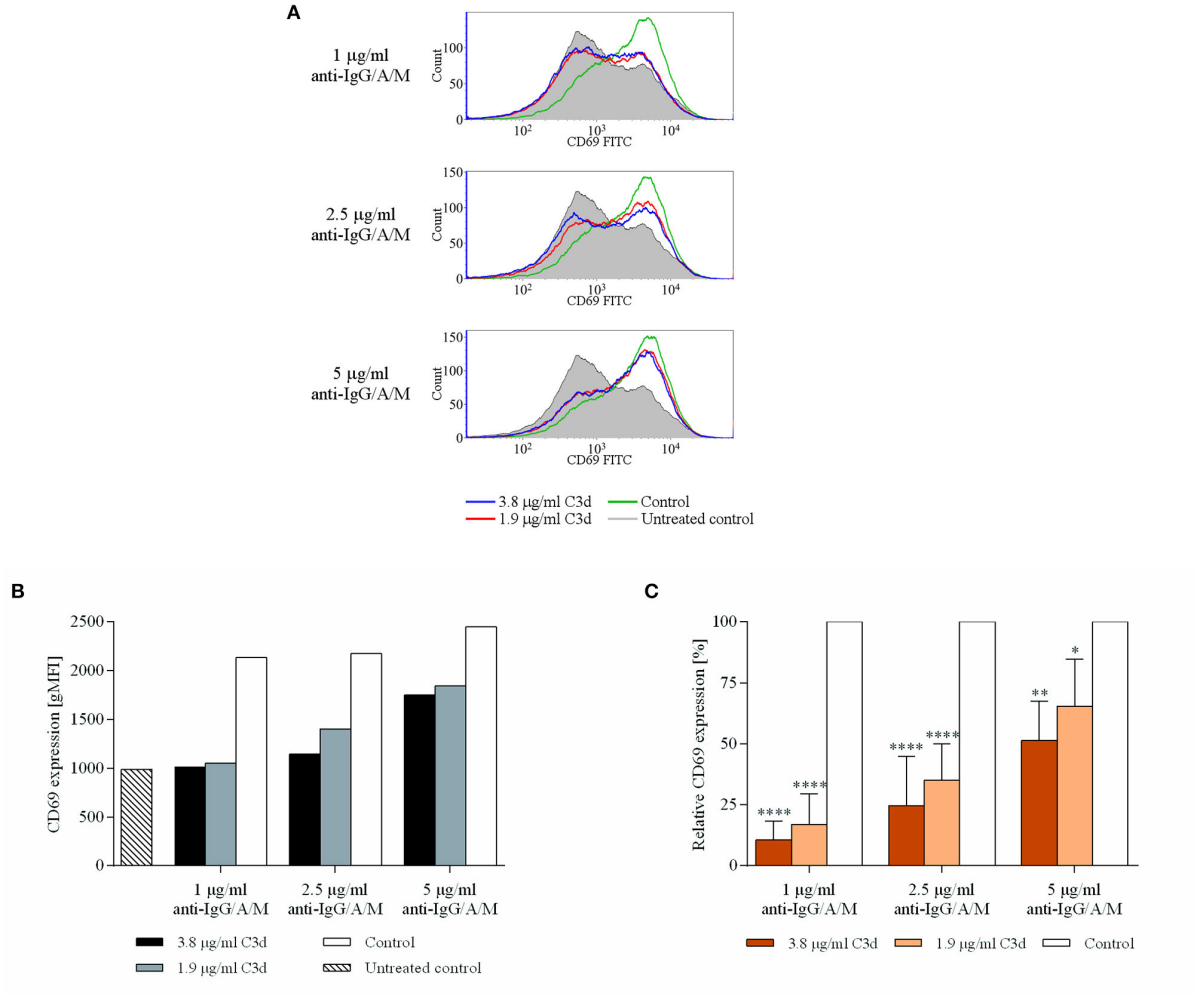
C3d inhibits the BCR-induced expression of CD69 activation marker, on human B cells. Resting human B cells were cultured with complexes consisting of either streptavidin-conjugated biotinylated anti-IgG/A/M (control) or streptavidin-linked biotinylated anti-IgG/A/M and biotinylated C3d in different concentrations as indicated. The expression of CD69 was measured by flow cytometry after 24 h. Histograms **(A)** and gMFI values **(B)** of a representative experiment are shown. Mean gMFI values of distinctly treated samples were compared to the control samples taken as 100%, and the cumulative results gained in three independent experiments display the mean of the relative values ± SD **(C)**. *p < 0.05, **p < 0.01, ****p < 0.0001.

CD69 represents the earliest marker of B-cell activation, which is expressed rapidly upon B-cell activation (23). It has recently been revealed that, apart from being an important marker to characterize the state of B cell activation, CD69 exerts regulatory functions on immune responses (24); therefore, its downregulation may affect further B cell functions, as well. Figure 2 shows that co-ligation of CR2 and BCR significantly and dose-dependently inhibited the appearance of CD69. The reduced expression is particularly noteworthy at low anti-IgG/A/M concentrations (1, 2.5 μg/ml) but is maintained even at higher BCR stimuli.

### C3d Inhibits BCR Induced IL-6 Production

Interleukin-6 is a proinflammatory cytokine secreted by numerous cell types, including B lymphocytes. This cytokine exerts a broad spectrum of autocrine and paracrine effects, affecting proliferation (25) and antibody production (26).

**Figure 3:**
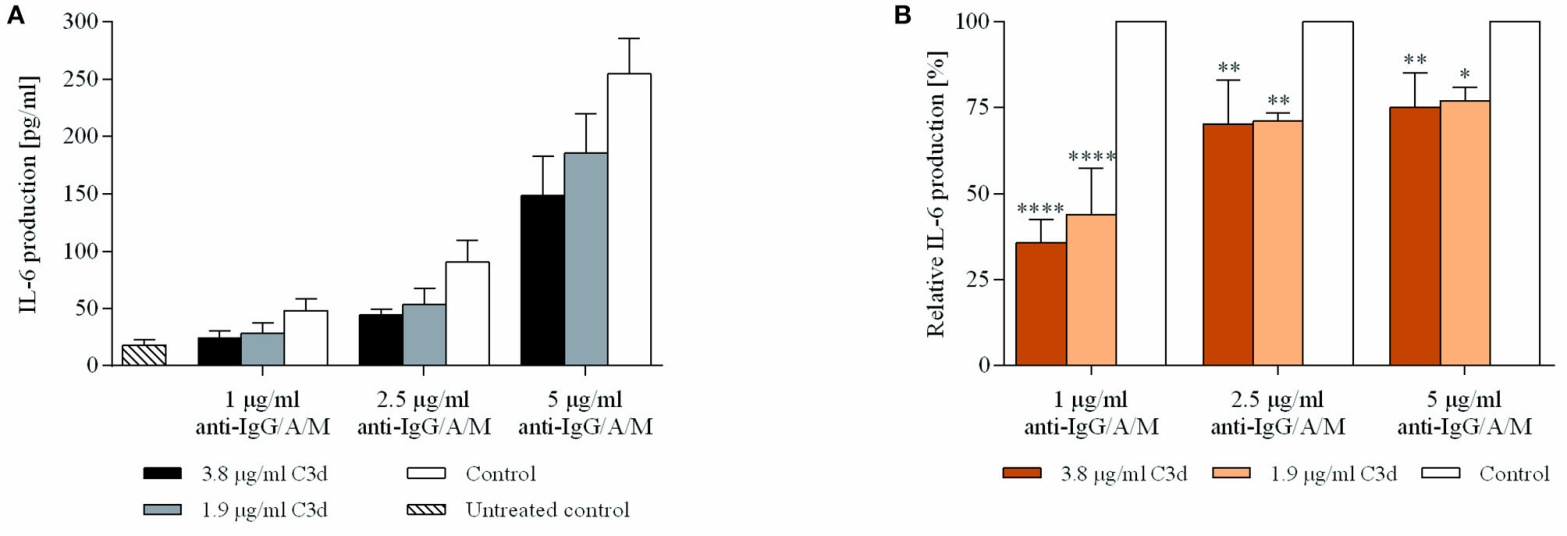
C3d suppresses the BCR induced IL-6 production of human B cells. Resting human B cells were cultured with complexes consisting of either streptavidin-conjugated biotinylated anti-IgG/A/M (control) or streptavidin-linked biotinylated anti-IgG/A/M and biotinylated C3d in different concentrations as indicated. Interleukin (IL)-6 production was measured by ELISA after 48 h. Mean IL-6 concentrations ± SD of triplicate samples of a representative donor are shown **(A)**. Mean IL-6 concentrations of differently treated samples were compared to the control samples taken as 100%. The cumulative results gained in three independent experiments display the mean of the relative values ± SD **(B)**. *p < 0.05, **p < 0.01, ****p < 0.0001.

Since we found that coclustering BCR and CR2 downregulates the expression of CD69 (Figure 2), we aimed to reveal how cytokine production is affected under the same conditions. Therefore, we treated resting human B cells with the preformed ligand complexes and assessed the IL-6 production after 48 h. We found that the coclustering of CR2 and BCR caused significant and dose-dependent suppression of IL-6 production, and the inhibition was the strongest in the case of a low BCR stimulus, similar to the decrease of the CD69 activation marker (Figure 3).

### C3d Inhibits BCR Induced Proliferation

Available data on the role of CR2 in the proliferative response of human B cells are contradictory. This might be due to the versatile experimental conditions, including the use of various B cell stimulatory agents ranging from pokeweed mitogen and Staphylococcus aureus Cowan I to B cell growth factor and anti-IgM (27–29), as well as employing different CR2 specific monoclonal antibodies (30, 31).

By applying conditions closer to the physiological system, we used C3d, the natural ligand of CD21, and triggered the BCR with the *F*(ab′)_2_ fragment of anti-IgG/A/M at suboptimal doses. Resting human tonsillar B cells were cultured with the preformed complexes of the ligands at various concentrations, and proliferation was assessed after 3 days.

**Figure 4:**
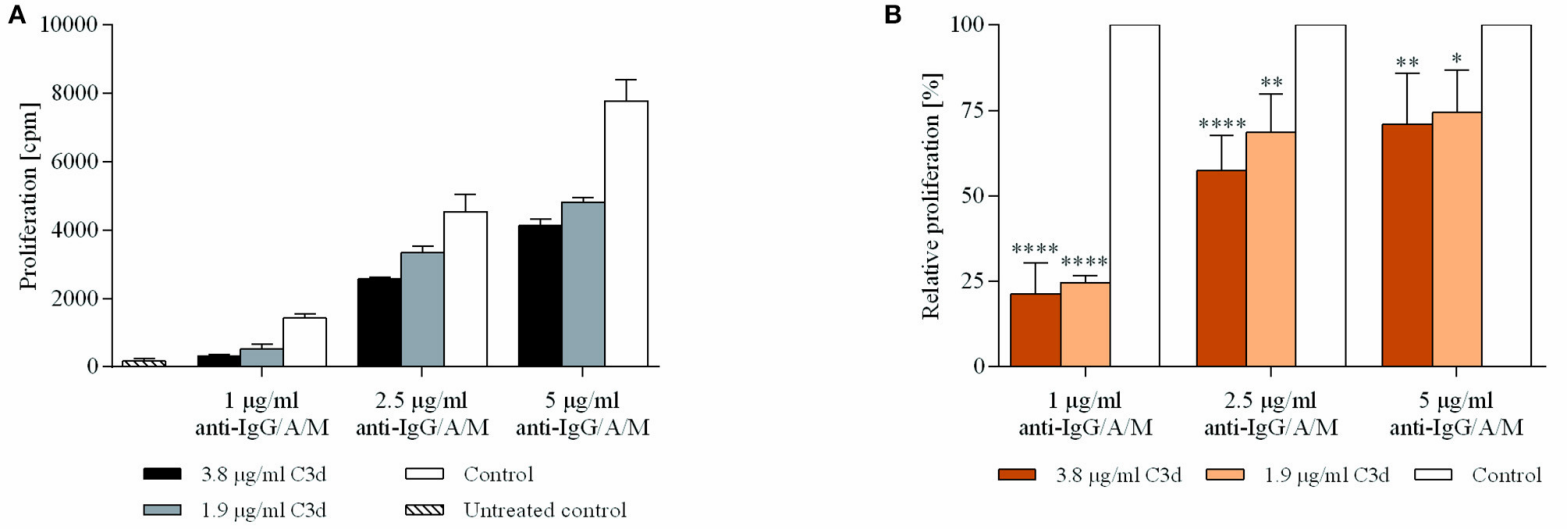
C3d inhibits the proliferation of BCR activated human B cells. Resting human B cells were cultured with complexes consisting of either streptavidin-conjugated biotinylated anti-IgG/A/M (control) or streptavidin-linked biotinylated anti-IgG/A/M and biotinylated C3d in different concentrations as indicated. Cells were pulsed with 1 μCi/sample [^3^H]-thymidine after 48 h and harvested 20 h later. Mean counts per minute (cpm) values ± SD of triplicate samples of a representative donor are shown **(A)**. The relative mean effect of C3d on proliferation ± SD in samples stimulated with BCR were calculated from three independent experiments **(B)**. *p < 0.05, **p < 0.01, ****p < 0.0001.

The results demonstrated that C3d exerts a significant and dose-dependent inhibitory effect on BCR-driven proliferation (Figure 4). The extent of the CR2-mediated inhibition indirectly correlated with the strength of the BCR stimulus.That is, at lower anti-BCR doses, C3d decreases proliferation by more than 75%, which gradually diminishes upon increasing the BCR stimulus.

C3d applied in the non-crosslinked form had no effect on the anti-IgG/A/M induced proliferation (data not shown).

### C3d Suppresses BCR Induced Antibody Production

Over the past 25 years, since the study in which C3d was designated as a molecular adjuvant enhancing antibody production in mice (32), there has been a remarkable absence of information regarding the impact of coclustering CR2 and BCR on antibody production by human B cells. Building on the results shown in Figures 1–4, we aimed to reveal how antibody production is influenced under similar conditions. To induce immunoglobulin production, IL-2, IL-6, and IL-10 were added to human B cells which were cultured in the presence of the ligand-complexes. After 3 days, cells were placed on plates coated with anti-IgM or anti-IgG, and the quantity of Ig-secreting cells was determined 20 h later.

**Figure 5:**
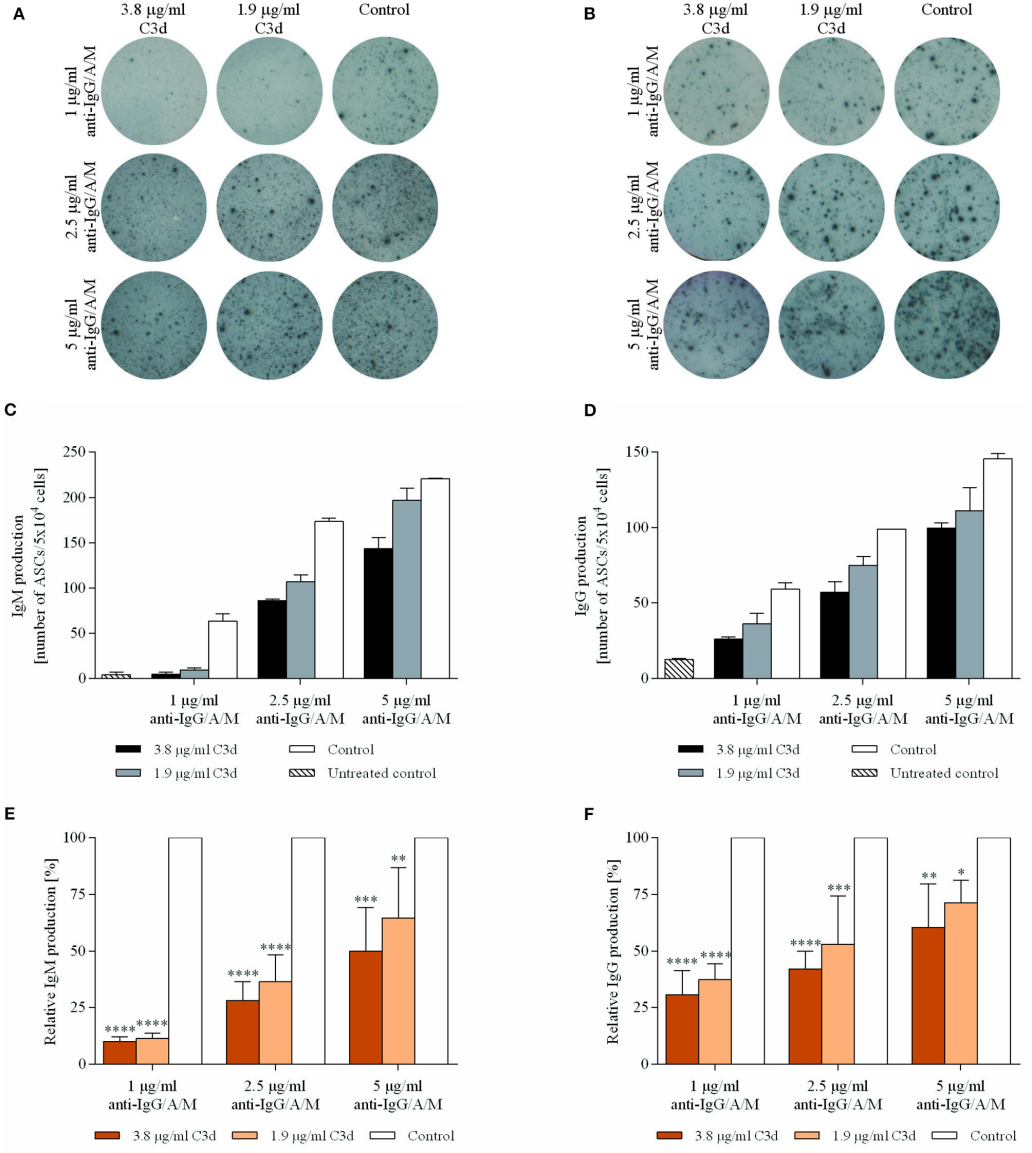
Antibody production is inhibited by co-ligation of CR2 with the BCR. Resting human B cells were cultured with complexes consisting of either streptavidin-conjugated biotinylated anti-IgG/A/M (control) or streptavidin-linked biotinylated anti-IgG/A/M and biotinylated C3d in different concentrations as indicated, for 72 h. IgM **(A,C,E)** and IgG **(B,D,F)** -antibody secreting cells (ASCs) were enumerated by enzyme-linked immunosorbent spot (ELISpot) assays. Images of representative membranes depicting the IgM **(A)** and IgG **(B)** producing B cells, and mean numbers of IgM **(C)** and IgG **(D)** secreting B cells ± SD of triplicate samples of a representative donor are shown. Quantity of IgM **(E)** and IgG **(F)** producing B cells was compared to the control samples taken as 100%. The cumulative results obtained in three independent experiments display the mean of the relative values ± SD. *p < 0.05, **p < 0.01, ***p < 0.001, ****p < 0.0001.

As shown in Figure 5, both IgM and IgG production were significantly and dose-dependently suppressed by the complexes. Again, the extent of inhibition was the strongest when the lowest suboptimal BCR stimulus was employed. The experiments carried out in the study revealed that, among the IgM^+^ B cells, C3d can evoke an almost complete inhibition of antibody production. The suppressive effect of CR2 ligation on the BCR induced antibody production was further strengthened when IgG production was assessed. The slightly reduced extent of inhibition observed in response to each level of BCR stimulus compared to IgM production might be due to the fact that IgG^+^ memory B cells differentiate into plasma cells rapidly after BCR activation, while IgM^+^ memory B cells reinitiate the germinal center reaction predominantly (33). Therefore, a similar level of BCR stimulation may provide a relatively stronger stimulus for IgG^+^ B cells, which cannot be counterbalanced by C3d as efficiently as in the case of IgM^+^ B cells.

The results unambiguously demonstrate that coengagement of BCR and CR2 significantly suppresses the antibody production of human B cells. Based on these findings and the lack of publications demonstrating that human CR2 acts as a coreceptor to elevate Ig-production, C3d cannot be regarded as a general molecular adjuvant which enhances the humoral immune response.

## Discussion

Since the early findings of Pepys (34), numerous studies have dealt with the role of complement in B cell functions. Subsequently, attention gradually turned toward C3d, which was proven to exert a significant enhancing effect on proliferation and antibody production of mouse B cells, both *in vitro* and *in vivo* (32, 35–37). Several researchers attempted to unveil a similar role for CR2 in human B cells, but the elevation of BCR-induced antibody production by C3d has not been demonstrated. Rightly, the tempting idea of C3d being a molecular adjuvant could not be developed to revolutionize human vaccination. Still, authors of several reviews generalized the results of mouse experiments to humans and defined CR2 as a coreceptor for BCR, whose eminent role is lowering the threshold for BCR stimulation. In the present study, we clearly demonstrated that human CR2 is an inhibitory coreceptor which mediates the suppression of several B-cell functions, including the expression of CD69, one of the very first phenotypic indicators of activation (Figure 2), the release of IL-6 (Figure 3), and proliferation (Figure 4) and antibody production (Figure 5) of human B lymphocytes.

We found an enhanced Ca^2+^ response of B cells triggered by C3d in the presence of suboptimal BCR stimulus (Figure 1), which does not initiate the nuclear factor-kappa B (NF-κB) signal transduction pathway, as reviewed by Scharenberg et al. (38). It has been demonstrated that a low but sustained level of Ca^2+^ response resulting from a weak BCR activation initiates only the nuclear factor of activated T-cell (NFAT) pathway, without the triggering of NF-κB signaling cascade. This leads to the downregulation of the expression of numerous immunoregulatory cell membrane molecules and cytokines, as well as to the suppression of B-cell proliferation and differentiation. Therefore, the initiation of the NFAT signaling pathway eventually induces and maintains B cell anergy, thereby playing a crucial role in the self-tolerance of B cells (39).

By attempting to utilize CR2, Carter et al. demonstrated a synergistic enhancement in the proliferation of human B cells after co-crosslinking BCR and CR2 (29), which was further supported by Mongini et al., who observed that co-ligation of BCR and CR2 significantly reduced the threshold for triggering proliferation in B cells (40). However, elevated antibody production was not demonstrated in either case. Furthermore, in these cumbersome studies, antibodies specific for BCR and for CR2 coupled with Sepharose and dextran, respectively, were applied, and consequently, these *in vitro* observations could never have been put into practice in the development of human vaccines. In functional experiments, we intended to apply conditions closer to the *in vivo* occurring situation; therefore, we used C3d, the natural ligand for CR2, and stimulated B cells suboptimally *via* their BCR (established in preliminary experiments). Moreover, we applied preformed receptor-ligand complexes, which mimic C3d-opsonized antigens or autoantigens and efficiently cluster BCR and CR2, which induced stimulatory effects on mouse B cells (37, 41, 42), underlying the validity of results of this study which demonstrate their opposite effects on human B cells.

The results obtained by studying CR2-deficient patients (43, 44) support the inhibitory role of CR1 in the regulation of human B cell functions. Thiel et al. found that neither the expression of CR1 (CD35) nor the activity of the complement system is impaired in the CD21-deficient patient (43); thus, complement-opsonized immune complexes are generated, and their binding to CR1 on B cells occurs. Consequently, the inhibitory function of CD35, proven by several studies (2–8), is maintained in patients with CR2 deficiency and most probably contributes to the diminished antibody response to pneumococcal polysaccharide vaccine (43). Furthermore, as reported by Wentink et al. (44), the reduced percentage of class-switched memory B cells and hypogammaglobulinemia as well as the impaired memory response to certain antigens described in patients with CR2 deficiency are most probably caused by the absence of CD21 on follicular dendritic cells, as CR2 is known to play crucial role in the germinal center reaction by trapping and retaining antigens on the surface of these cells.

**Figure 6:**
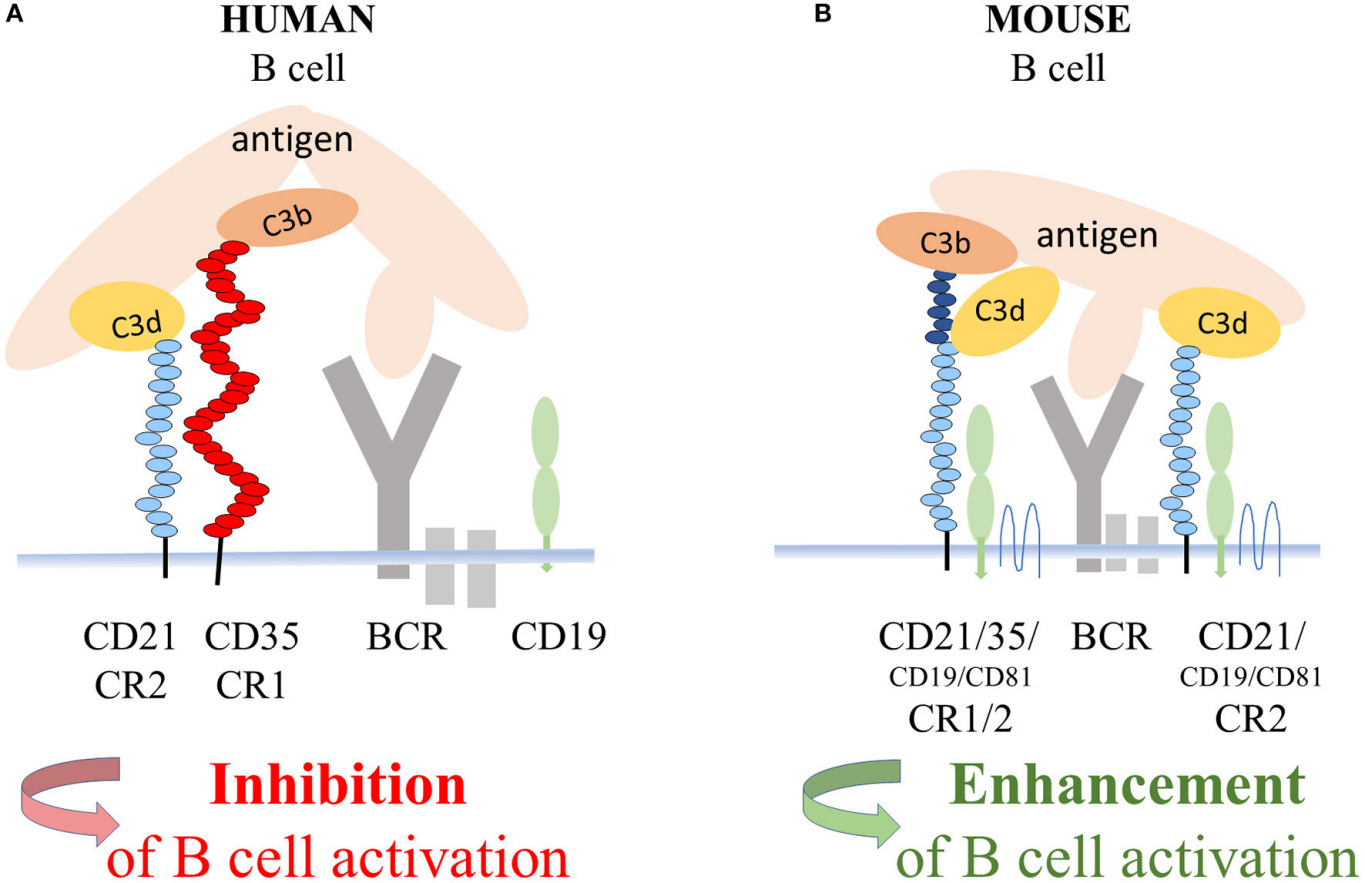
Suggested mechanism of the CD21 mediated inhibition of BCR triggered functions of human B cells. In the coclustered complexes of human CD21 and CD35, from which CD19 is excluded, the strong inhibitory effect of CR1 controls BCR-induced activation **(A)**. In mice, coclustering BCR and the trimolecular complex containing CD19 and CD81 and the alternatively spliced CR1/2 enhance B-cell activation **(B)**.

Regarding the mechanism of the CD21-mediated inhibition of human B cell functions, the most likely scenario is that it is exerted by the complex of CR1 and CR2 (Figure 6), whose presence in the membrane of human B lymphocytes had been demonstrated earlier by Tuveson et al. (16). This study also showed that CD19, the important signal transducing molecule, is excluded from this complex since the interaction of CR2 with CR1 prevents the association between CR2 and CD19. Further along this line, the strong suppressive effect of human CR1 on BCR-mediated functions has been extensively proven *in vitro* as well as *in vivo* (2–8). Ligation of CR1 has been shown to inhibit BCR-induced phosphorylation of Syk, which is essential for B cell functions (6). Thus, it is plausible to assume, that the CR2-mediated inhibition of the BCR induced proliferation and antibody production is due to the association between CR2 and the inhibitory CR1. A number of CR2 molecules appear on the surface of human B cells in association with CD19 (16), which might enhance B-cell activation and antibody production under certain conditions. The signal transducing activity of this complex, however, was shown to be inactivated upon ligation of surface Ig of human B cells (45), which further strengthens our results. Furthermore, by comparing a patient with CD21 deficiency and a patient with CD19 and CD81 deficiency, Wentink et al. concluded that the observed hypogammaglobulinemia in the latter case could be explained by defective BCR signaling, which is normal and intact in CD21 deficiency (44). Thus, the absence of CD21 does not lead to impaired BCR signaling, which strongly supports the finding that, in human, the role of CR2 is not reducing the threshold of BCR activation.

Since CR2 binds self-antigens, such as ssDNA, dsDNA, chromatin, and histones (46), the results shown in this study raise the possibility that, in human, CD21 participates in the maintenance of peripheral B-cell tolerance in a, so far, undescribed manner, by suppressing the response of autoreactive B lymphocytes.

In conclusion, the “textbook dogma” claiming that CR2 is a positive coreceptor for the BCR in general, must be revised. The significant differences between the genetic background of CR1 and CR2 in human and mice led to profoundly different functions and opposite effects in these two species when these two complement receptors are coengaged with the BCR. While antibody production is enhanced in mice, it is suppressed in human. Therefore, despite several experimental capabilities and advantages, mouse systems are far from being optimal for studying the role of complement in the function and manipulation of human B cells.

## Data Availability Statement

The raw data supporting the conclusions of this article will be made available by the authors, without undue reservation.

## Ethics Statement

The studies involving human participants were reviewed and approved by the Ethics Committee of the Medical Research Council in Hungary (TUKEB), 52088/2015/EKU. Written informed consent to participate in this study was provided by the participants' legal guardian/next of kin.

## Author Contributions

AE, KK, and BM-V designed the study and wrote the manuscript. KK and BM-V performed the experiments. KK, BM-V, JM, ZB, and AE analyzed the data. AE supervised research. All authors contributed to the article and approved the submitted version.

## Conflict of Interest

The authors declare that the research was conducted in the absence of any commercial or financial relationships that could be construed as a potential conflict of interest.
