# Supplementary material for: Revisiting the Coreceptor Function of Complement Receptor Type 2 (CR2, CD21); Coengagement With the B-Cell Receptor Inhibits the Activation, Proliferation, and Antibody Production of Human B Cells
Source: Front Immunol. 2021 Apr 1;12:620427. doi: 10.3389/fimmu.2021.620427 (PMC8047317; doi:10.3389/fimmu.2021.620427)
Supplement: Supplementary file 1 [file Data_Sheet_1.PDF]

Supplementary Material

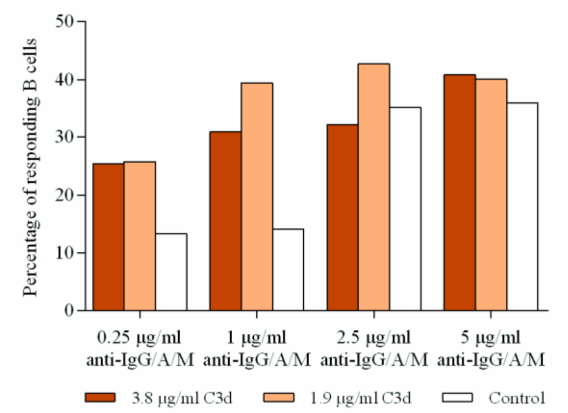

Supplementary Figure 1. Co-ligation of CR2 with suboptimally stimulated BCR enhances the Ca^{2+} response of human B cells

Resting human B cells were treated with complexes consisting either exclusively of streptavidin conjugated anti-IgG/A/M (control samples) or streptavidin linked anti-IgG/A/M and C3d molecules in concentrations as indicated. Percentage of the B cells responding with Ca^{2+} response to the particular treatments is shown in one representative experiment.
